# Experiences of psychosomatic symptoms and self-management strategies among patients with advanced lung cancer undergoing chemotherapy: a qualitative study based on symptom management theory

**DOI:** 10.3389/fpubh.2026.1807299

**Published:** 2026-03-27

**Authors:** Bangyan Xiong, Chan Jin, Zhouyang Zhang, Renhua Xu

**Affiliations:** School of Nursing, Binzhou Medical University, Yantai, China

**Keywords:** advanced lung cancer, chemotherapy, psychosomatic symptoms, qualitative research, symptom management theory

## Abstract

**Background:**

Lung cancer is among the malignancies with the heaviest disease burden worldwide. Patients with advanced lung cancer face substantial physical and psychological challenges during chemotherapy-based treatment. Psychosomatic symptoms are important factors influencing patients’ quality of life and treatment adherence. However, although the incidence of psychosomatic symptoms in patients with advanced lung cancer undergoing chemotherapy is relatively high, their identification rates remain low.

**Objectives:**

This study guided by symptom management theory, and aims to explore from the patients’ perspective, the psychosomatic symptom experiences, management strategies, outcomes and influencing factors among individuals undergoing chemotherapy for advanced lung cancer.

**Methods:**

A descriptive qualitative research design was employed. Using purposive sampling, semi-structured interviews were conducted with 17 patients undergoing chemotherapy for advanced lung cancer in the Department of Medical Oncology at a tertiary Grade A hospital in Yantai City between September and December 2025. Data were analyzed using directed content analysis, and themes were extracted accordingly. NVivo 15.0 software was utilized for data management and coding.

**Results:**

After organizing, analyzing, and synthesizing the interview data, four themes and twelve categories emerged from the qualitative data: symptom experiences(complex and diverse physical symptom experiences, multidimensional interaction of psychological symptom experiences, limited social roles and functional capacity); symptom management strategies(positive approach to challenges, negative responses to symptoms); symptom outcomes(persistent psychological burden and disease-related uncertainty, reconstructing psychosomatic adaptation and self-management abilities, seeking professional medical assistance and psychological support); influencing factors(individual factors, disease and treatment factors, family and social environmental factors, cultural factors).

**Conclusion:**

Based on symptom management theory, this study found that patients with advanced lung cancer undergoing chemotherapy experience multidimensional and complex psychosomatic symptoms, display varying coping strategies and have diverse symptom outcomes. Their symptom management is influenced by multiple factors, including individual characteristics, environmental conditions, and disease-related aspects. Healthcare professionals should closely attend to patients’ psychosomatic experiences, guide them in adopting positive coping strategies, facilitate accurate disease understanding, and promote psychological well-being. Simultaneously, identifying the relevant factors affecting symptom management and providing targeted health education can help improve long-term symptom outcomes and reduce the overall disease burden.

## 1 Introduction

Lung cancer ranks first in both incidence and mortality among all malignant tumors in China (1), representing a significant threat to patients’ survival and overall health. Most patients are diagnosed at locally advanced or metastatic stages, at which point curative surgery is no longer feasible, and the five-year survival rate is approximately 20% (2). Chemotherapy is one of the important treatment modalities that can significantly improve patients’ prognosis and is widely implemented in clinical practice (3). Evidence from previous studies suggests that patients with advanced lung cancer receiving chemotherapy frequently experience a spectrum of psychosomatic symptoms. These include physical manifestations, such as fatigue, nausea, vomiting, and sleep disturbances, as well as psychological symptoms, including anxiety, depression, and fear (4–8). These symptoms stem not only from the tumor itself but are also closely associated with factors such as the toxic effects of chemotherapy, treatment schedules, and the inherent uncertainty of the disease. The interaction between psychological and physical symptoms can undermine treatment adherence and substantially reduce patients’ quality of life (9, 10). Research found that patients adopt different coping strategies when facing symptom distress (11, 12). Those who employ positive coping approaches are more likely to alleviate symptom-related distress by adjusting their lifestyle, seeking support from family and society, and obtaining professional guidance from healthcare providers. Coping refers to an individual’s response to stress, emotions, problems, and difficult situations (13). Meneguin et al. (14) found that lung cancer patients with higher levels of self-efficacy were more likely to adopt proactive health behaviors and coping strategies, thereby enabling more effective management of disease-related symptoms. Self-efficacy refers to an individual’s confidence or belief in their ability to perform specific tasks successfully and achieve desired outcomes. Patients with high self-efficacy are more likely to actively engage in health behaviors and confront their fears, whereas those with low self-efficacy tend to avoid them (15).

To support symptom management in patients, some studies have implemented psychological and behavioral interventions, such as psychoeducation (16), managing cancer and living meaningfully therapy (17), magnanimous therapy (18), and music therapy (19), etc. These approaches help patients adjust their cognition, restructure self-identity, and cultivate positive emotional and behavioral traits. Exercise-based interventions, including Baduanjin and resistance training (20), have also been shown to improve patients’ quality of life, alleviate depression and anxiety, reduce fatigue, and enhance sleep quality. Furthermore, some studies have employed emerging technologies, such as wearable devices integrated with artificial intelligence algorithms (21), mobile healthcare platforms (22), and virtual reality-supported acceptance and commitment therapy (23), to remotely monitor patients’ symptom changes and provide remote care, helping to alleviate symptom distress.

Previous studies (3, 10–12, 14) have primarily employed quantitative methods to investigate the relationships between symptoms experienced by patients with advanced lung cancer undergoing chemotherapy and their associated influencing factors. At present, assessment largely relies on a range of psychological and symptom evaluation scales. Although these instruments include items related to both physical symptoms and psychological states, they are neither comprehensive nor specifically tailored for patients receiving chemotherapy for advanced lung cancer. Given the subjective and multidimensional nature of these symptoms, sole reliance on scales or questionnaires to assess patients’ experiences presents inherent limitations.

Qualitative research is particularly valuable for gaining an in-depth understanding of patients’ symptom experiences and coping strategies. Such approaches have been widely applied to explore symptom experiences in other disease contexts (24–26). However, qualitative research focusing on patients with advanced lung cancer undergoing chemotherapy remains limited, particularly with respect to the systematic exploration of integrated psychosomatic symptom experiences and self-management strategies (27). Symptom Management Theory (SMT) (28, 29) emphasizes that all symptoms require active management, with the overarching goals of reducing symptom burden, improving clinical outcomes, and enhancing patients’ quality of life. It posits that an effective symptom management process consists of three core components: symptom experience, symptom management strategies and outcomes. Which integrates people, environment, health and disease, and emphasizes the criticality of how to perceive and manage symptoms during an individual’s illness (30). This theory has been applied in symptom management research among cancer patients, individuals with patent foramen ovale, patients with corneal ulcers, kidney transplant recipients, and other populations (24–26, 31).

However, no previous study has applied symptom management theory to explore the psychosomatic symptom experiences, management strategies, outcomes, and influencing factors among patients with advanced lung cancer undergoing chemotherapy. Therefore, this study utilized SMT as a framework to explore these aspects in depth through qualitative research methods. The findings aim to provide medical staff with a deeper understanding of patients’ experiences, offer evidence-based approaches for symptom management from multiple perspectives, and serve as a reference for implementing more effective clinical interventions.

## 2 Methods

### 2.1 Design

The study utilized a descriptive qualitative research design, and the research team adhered to the Consolidated Criteria for Reporting Qualitative Research (COREQ) (32) to ensure compliance with established standards for reporting qualitative data (see Supplementary file 1).

### 2.2 Study setting and recruitment

The purposive sampling was employed to recruit patients with advanced lung cancer undergoing chemotherapy from the oncology department of a tertiary hospital in Yantai between September and December 2025. Sampling was guided by the principle of maximum variation, taking into account factors such as gender, age, educational level, and time since diagnosis. Inclusion criteria were as follows: (1) patients with pathologically confirmed stage III or IV lung cancer undergoing chemotherapy, having completed treatment at least three days prior, with an estimated survival of ≥6 months; (2) age ≥18 years; (3) awareness of their diagnosis, disease status, and treatment plan; (4) Informed consent for the study; (5) ability to communicate effectively. Exclusion criteria included: (1) presence of other malignant tumors or psychiatric/psychological disorders; (2) serious hearing, speech, and cognitive dysfunction; (3) concurrent participation in other studies. The interview sample size was guided by the principle of information saturation, defined as the point at which no new themes emerge during data analysis (33).

### 2.3 Interview outline

This study employed semi-structured interviews, and the interview outline was developed based on SMT. First, the research team systematically searched for and reviewed relevant literature on the experiences and management of psychosomatic symptoms in patients with cancer to identify the core keywords related to the research topic of this study. Second, guided by the SMT framework, the research team initially developed an interview guide based on dimensions such as psychosomatic symptom experiences, symptom management strategies, symptom outcomes, and influencing factors. The research team also conducted multiple rounds of discussion and revision to refine the content and wording of the interview questions. Subsequently, one expert in psychosomatic medicine and two oncology experts were invited to review the interview outline and provide suggestions regarding the appropriateness and comprehensiveness of the questions. Finally, pre-interviews were conducted with two patients undergoing chemotherapy for advanced lung cancer. Based on the results of these pre-interviews, the wording of certain questions was appropriately refined. The final interview outline was established, and data from the pre-interviews were not included in the final research findings. The final interview outline is presented in Table 1.

**Table 1:** Interview outline of this study.

SMT	Interview content
Symptom experiences	What physical discomforts have you most frequently experienced during the course of your illness and chemotherapy?
	In addition to physical symptoms, what noticeable changes have you experienced in your emotional or psychological state?
	How have these physical and psychological symptoms affected your daily life?
Symptom management strategies	When these symptoms become distressing or difficult to tolerate, what strategies do you usually adopt to relieve or cope with them?
Symptom outcomes	How effective do you perceive these strategies to be?
Influencing factors	Have you received support from family members, friends, or medical staff in managing your symptoms? If so, what forms of support were provided?
	What factors do you believe exacerbate or alleviate your psychosomatic symptoms?
	In what areas do you hope to receive additional support or assistance from medical staff?

### 2.4 Data collection

Data were collected through face-to-face, semi-structured in-depth interviews. The interviews were conducted in a quiet and comfortable demonstration classroom within the oncology department, free from interruptions. Throughout the data collection process, the researcher adhered strictly to principles of anonymity and confidentiality. Before the interview, the researcher explained in detail the purpose, content, and methods of the interview to the participants, while also gaining their trust and establishing a positive nurse–patient relationship. Written informed consent was obtained from all participants, and interview times were scheduled in advance. During the interviews, the interviewer maintained a neutral stance and flexibly adjusted the questioning sequence and approach according to participants’ responses and emotional states. Participants were encouraged to express their genuine feelings in a relaxed atmosphere. All interviews were audio-recorded, and non-verbal cues, including participants’ gestures, facial expressions, and behaviors, were observed and documented. Key points and interpretations were verified with participants in a timely manner to enhance data accuracy. At the end of the interviews, the participants were given a brief reminder, and the interviews were concluded. Only the interviewer and the participant were present during each interview. Each interview was conducted for 30–45 min, depending on the individual circumstances. All invited participants completed the study. No repeat interviews were conducted, and no participants withdrew during the study.

### 2.5 Data analysis

Following each interview, the researchers transcribed the audio recordings and interview notes verbatim within 24 h and subsequently reviewed the transcripts for accuracy. A second researcher then cross-checked the transcripts to ensure completeness and reliability of the data. Verified transcripts were returned to participants for member checking prior to data analysis. Each participant was assigned a unique code (P1–P17), and individual Word documents were created for each interview. NVivo 15.0 software was used to organize, store, and manage the qualitative data. To align with symptom management theory and extend its application to patients with advanced lung cancer undergoing chemotherapy, interview data were analyzed using directed content analysis (34). The procedure was as follows: (1) Key concepts were identified from SMT and previous literature (24–26) to establish preliminary coding categories. (2) The transcripts were read repeatedly, and statements closely related to the research questions were identified and highlighted. (3) Relevant semantic units were categorized according to the predetermined codes, with new codes created for content that did not fit existing categories. The original coding framework was revised concurrently, resulting in new categorical attributes that expanded SMT.(4) Categories and subcategories were constructed based on relationships among codes. (5) The results were interpreted and analyzed, linking findings back to the original data, with representative excerpts selected as illustrative examples. During the analysis process, any discrepancies in coding or theme interpretation were resolved through group discussion to reach consensus.

The research team consisted of one professor and three postgraduate students in nursing, all of whom had received training in qualitative research methods, the first author (BX) is a female. BX conducted the interviews and recorded field notes, while ZZ was responsible for verifying the collected data. Data analysis was independently performed by BX and CJ, who resolved differences through discussion. A third researcher RX was consulted to review and arbitrate the research findings, ensuring rigor and reliability in the analysis process.

### 2.6 Ethical considerations

This study has been approved by the Ethics Committee of Yantai Affiliated Hospital of Binzhou Medical University (Ethical Approval Number: 20250908140).

### 2.7 Rigor and reflexivity

We used the qualitative research criteria proposed by Lincoln and Guba to enhance the trustworthiness of this study (35). The interviewer was a master’s-level nursing student with more than five years of clinical nursing experience who had received systematic training in qualitative research theory and methodology, demonstrating strong proficiency in qualitative research techniques and interviewing skills. No prior relationship existed between the researchers and the participants before the study. Throughout the research process, the investigator engaged in ongoing self-reflection to minimize the potential influence of professional background and personal values on data collection and analysis. One member of the research team transcribed the interview recordings verbatim, while other members conducted cross-checks to ensure data accuracy. During the interview, the interviewer promptly followed up with the interviewee on unclear or questionable points or repeated them back to the interviewee for confirmation. During the data coding and analysis process, the researchers maintained a neutral stance and avoided introducing personal value judgments. Two researchers independently analyzed the data, and discrepancies were subsequently discussed within the research team until consensus was reached on the final coding framework and themes, thereby enhancing the rigor and credibility of the study.

## 3 Results

**Figure 1:**
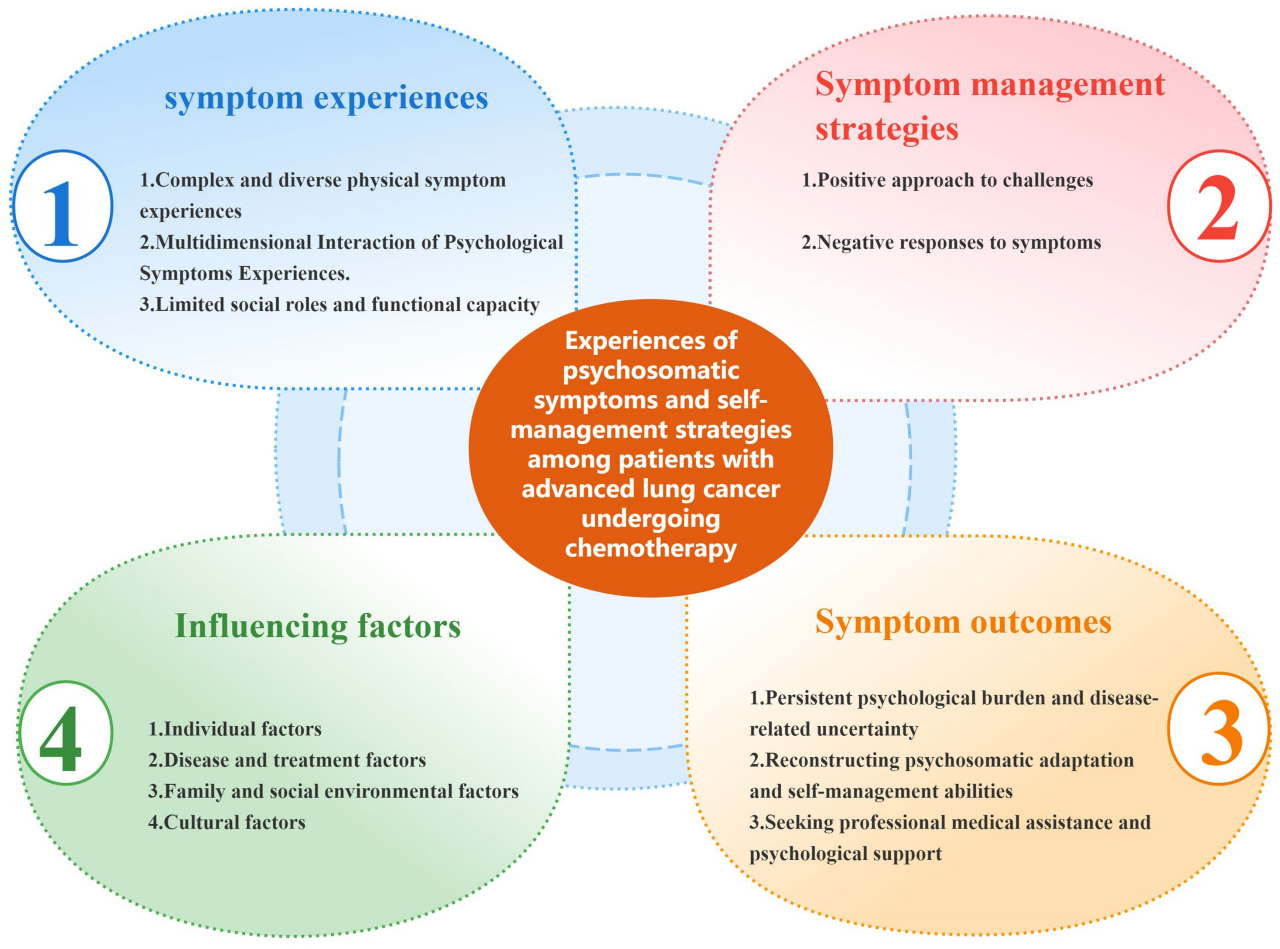
Symptom experiences, management strategies, outcomes and influencing factors of patients with advanced lung cancer undergoing chemotherapy.

After interviewing the 15th participant, no additional information was obtained, and two more participants were recruited to ensure data saturation. The final sample included 17 participants, comprising 12 males and 5 females, with ages ranging from 43 to 73 years (mean age: 62.65 ± 7.66 years). Detailed demographic characteristics are presented in Table 2. Guided by symptom management theory, this study identified four main themes and twelve sub-themes (Figure 1).

**Table 2:** The characteristics of interviewees (*n =* 17).

Number	Sex	Age	Pathological types	Duration of diagnosis (months)	Occupation	Education background	Monthly income, CNY	Stage of cancer	Medical insurance type
P1	Male	65	SCLC	>12	Farmer	Primary school	<3,000	IV	Rural medical insurance
P2	Female	51	NSCLC	>12	Service	University	3,000–6,000	IV	Urban medical insurance
P3	Male	70	SCLC	>12	worker	High school	3,000–6,000	IV	Urban medical insurance
P4	Female	68	NSCLC	>12	Farmer	Middle school	<3,000	IV	Rural medical insurance
P5	Female	59	NSCLC	>12	worker	Middle school	<3,000	III	Urban medical insurance
P6	Male	69	NSCLC	>12	worker	Middle school	<3,000	III	Urban medical insurance
P7	Male	66	NSCLC	>12	worker	High school	3,000–6,000	IV	Urban medical insurance
P8	Male	63	NSCLC	>12	Farmer	Middle school	<3,000	IV	Rural medical insurance
P9	Male	69	SCLC	6–12	worker	Middle school	<3,000	IV	Urban medical insurance
P10	Male	54	NSCLC	>12	Self-employed	High school	3,000–6,000	III	Urban medical insurance
P11	Male	65	SCLC	6–12	worker	Middle school	<3,000	IV	Rural medical insurance
P12	Male	73	NSCLC	>12	Service	University	>6,000	IV	Urban medical insurance
P13	Female	65	SCLC	<6	Farmer	Middle school	<3,000	III	Rural medical insurance
P14	Male	59	NSCLC	>12	Service	High school	3,000–6,000	IV	Urban medical insurance
P15	Male	43	NSCLC	>12	Self-employed	University	3,000–6,000	IV	Urban medical insurance
P16	Male	60	SCLC	<6	Farmer	Middle school	<3,000	III	Rural medical insurance
P17	Female	66	NSCLC	>12	worker	Middle school	<3,000	IV	Urban medical insurance

Small cell lung carcinoma is represented by (SCLC), and non-small-cell lung carcinoma is represented by (NSCLC).

### 3.1 Symptom experiences

#### 3.1.1 Complex and diverse physical symptom experiences

##### 3.1.1.1 Cough and sputum production

Respiratory symptoms were among the most frequently reported physical discomforts. Seven participants described persistent coughing and sputum production, which interfered with daily communication and sleep quality.

“The main symptom is coughing. I came to the hospital because of the cough. It’s very severe, and I can’t sleep at night” (P9).

“I have a cough and produce yellow sputum. The phlegm is quite sticky, as if something is lodged deep in my throat, and I have to exert considerable effort to cough it up” (P14).

##### 3.1.1.2 Chest tightness, shortness of breath, and breathing difficulties

Nine participants reported experiencing chest tightness, shortness of breath, and breathing difficulties. These symptoms were often exacerbated at night, after physical activity, or during periods of emotional stress, negatively impacting daily functioning and sleep.

“I feel breathless. Even a slight movement makes me feel out of breath. When I walk from the ward to the other end of the corridor, I must stop and rest for a while.” (P8).

“The most obvious physical symptom is shortness of breath and chest tightness. Sometimes I can’t catch my breath, especially when I sleep at night. As soon as I lie flat, my chest feels blocked, and I can only sit up to breathe.” (P10).

##### 3.1.1.3 Pain

Pain was a commonly reported symptom during the course of the illness and treatment. Participants described pain occurring in different body locations and varying in intensity, often attributing it to tumor progression or metastasis.

“The most noticeable symptom is a sore throat. I feel it when eating, drinking, or talking. Even if I try to ignore it, I just can’t” (P7).

“(Pointing to the leg) The most uncomfortable part is my leg. When it hurts, even walking a short distance becomes unbearable. You can even touch a hard lump on the bone” (P6).

##### 3.1.1.4 Fatigue and weakness

Twelve participants reported experiencing fatigue and physical weakness following chemotherapy, which limited their ability to perform daily activities and engage in self-care.

“I always feel tired. Even after getting up in the morning, I still feel very exhausted, as though I haven’t rested at all. Many times, I haven’t done anything, yet I still feel exhausted. When I’m sitting, I want to lie down, but after lying down, I feel uncomfortable” (P16).

“I feel extremely weak, with no energy at all. Even simple tasks feel exhausting. Things that used to be easy to pick up now feel very heavy in my hands. I can’t hold them for long before my arms start to ache, so I have to put them down and rest. Sometimes I want to do small chores at home, but I quickly feel overwhelmed. Standing for a while makes me tired, and even walking a few steps requires me to stop and take a break. The doctor said this is a side effect of the chemotherapy drugs” (P5).

##### 3.1.1.5 Sleep disturbances

Fourteen participants reported experiencing sleep-related problems, including difficulty initiating sleep, frequent nocturnal awakenings, and poor overall sleep quality.

“I can’t sleep soundly. I often wake up before dawn, and once I’m awake, my mind becomes completely alert. No matter how I lie down, I just can’t fall back asleep, I can only endure it” (P14).

“I couldn’t sleep well in the hospital. It was very uncomfortable and irritating. At home, it was a bit better. On the one hand, there were many people in the hospital, and it was noisy. On the other hand, during hospitalization, I didn’t feel at ease” (P2).

##### 3.1.1.6 Gastrointestinal discomfort

Fifteen participants reported experiencing gastrointestinal symptoms, including decreased appetite, nausea, vomiting, acid reflux, and other forms of digestive discomfort. These symptoms affect dietary intake and nutritional absorption to varying degrees, particularly during and shortly after chemotherapy.

“The chemotherapy period was the most unbearable. I felt nauseous, bloated, and kept belching. My husband cooked me a bowl of noodles, but after only a few bites, my stomach began to feel uncomfortable, I kept belching, and a sour taste slowly rose in my throat, so I had to put my chopsticks down” (P2).

“The days immediately after chemotherapy were especially difficult, severe nausea and vomiting. Even the faint smell of cooking oil made me feel sick, and I had no appetite at all” (P15).

##### 3.1.1.7 Hair loss and changes in appearance

Three participants reported distress related to hair loss and other treatment-induced physical changes. Concerns about bodily alterations and heightened sensitivity to others’ reactions negatively affected their self-image.

“The chemotherapy drugs really took a toll on me. My hair almost completely fell out. Look at my head now completely shaved. A few days ago, I even noticed dark spots on my forehead when I looked in the mirror. It looks terrible” (P16).

“During that period, I lost about seven or eight pounds. One day, I noticed in the mirror that my face looked much thinner than before, and my clothes felt looser on my body. I realized how much weight I had lost, and it made me feel a little heavy inside” (P12).

##### 3.1.1.8 Other physical symptoms

In addition to the commonly reported symptoms, participants experienced other physical discomforts such as tinnitus, palpitations, and dizziness.

“I also had tinnitus, a constant buzzing in my ears, like cicadas chirping. It became particularly noticeable in the last month, sometimes so loud that it made me extremely upset” (P7).

“Sometimes I felt my heart racing, like it was beating faster than usual. I had an electrocardiogram (ECG), and the doctor told me everything looked normal. Although the test showed no problems, the sensation of my heart pounding still made me uneasy at times” (P17).

“(Sighs softly) The most common symptom… is dizziness. My head often feels heavy and sluggish. Sometimes, when I stand up or walk, I feel a little unsteady, and I worry I might lose my balance and fall. I’m extra careful because I’ve fallen once before, and I don’t want it to happen again” (P1).

#### 3.1.2 Multidimensional interaction of psychological symptom experiences

Confronted with the prolonged and recurrent distress caused by the disease, patients often experience a range of psychological symptoms, including anxiety, depression, fear, and self-blame. These symptoms tend to intensify over time and may become internalized, contributing to persistent psychological burden. Although patients may appear composed in front of others, their true emotions are frequently suppressed, gradually forming a hidden and enduring psychological strain.

##### 3.1.2.1 Anxiety and depression

Thirteen participants reported experiencing persistent low mood and loss of interest during prolonged treatment and symptom recurrence, often choosing to cope with these emotional fluctuations alone.

“In my heart… sigh… I can’t put it into words, but sometimes, when everything is fine and no one is bothering me or teasing me, a heavy feeling suddenly comes over me, extremely depressing. Other times, I look out the window, and tears just fall uncontrollably” (P5).

“I live alone, so no matter what happens, I have to deal with it by myself. Whether I’m feeling unwell or need to go to the hospital, there’s usually no one around to help. Most of the time I tell myself I can manage, but sometimes I still feel incredibly low” (P15).

##### 3.1.2.2 Fear and dread

All participants reported experiencing intense fear and dread related to their illness, treatment, and future prognosis. These emotions were severe at the time of initial diagnosis, during symptom exacerbation, or while awaiting follow-up examinations.

“Right after I was diagnosed with lung cancer, I completely broke down. When the doctor said ‘cancer,’ I couldn’t sleep at all during that period, overwhelmed by intense fear and anxiety. I aways felt like I didn’t have much time left” (P10).

“(Sigh) To be honest, when I first heard I had this disease (lung cancer). I was extremely scared and had no idea what to do. At that moment, my mind went completely blank. I hardly paid attention to what the doctor said afterward and just nodded mechanically” (P2).

##### 3.1.2.3 Irritability and heightened anger

Ten participants reported decreased emotional stability, becoming more prone to negative reactions in response to minor daily events or interactions with others.

“Sometimes I get extremely anxious. I already have a quick temper, and now I tend to lose my temper more easily. For example, the intravenous infusion is slow or the food is cold, I tend to get angry” (P11).

“During the days after chemotherapy, my reactions were particularly intense. If I didn’t sleep well at night, I would feel extremely irritable the next day. Even small things could set me off, and I found myself losing patience easily” (P14).

##### 3.1.2.4 Loss of control and frustration

Eleven participants described feelings of powerlessness in coping with the physical changes and limitations brought on by their diagnosis and chemotherapy. These experiences were accompanied by a perceived loss of control over their bodies and daily lives, leading to frustration and emotional distress.

“Before, I was a team leader at work, and everyone in the office followed my instructions. Now that I’m ill, I must take medicine, receive injections, and attend check-ups; all according to others’ directions. Sometimes I feel a ‘being arranged’ sensation in my heart, and this sense of disparity is quite uncomfortable” (P12).

“Now even carrying a bucket of water is exhausting for me. How could I not feel frustrated? A grown man reduced to this; it’s an indescribable pain” (P16).

##### 3.1.2.5 Feelings of pressure and helplessness

Eleven participants reported experiencing stress stemming from multiple aspects of their illness and daily life, often accompanied by a sense of helplessness.

“When I’m upset, my whole body feels uncomfortable, and I can’t really explain what’s wrong. There’s no one who can help me with that feeling, so I can only bear it by myself” (P13).

“Sometimes I really want to talk more with my children, but they are always very busy. When I call them, we can only talk for a few minutes before they hang up, which makes me feel a bit empty inside” (P8).

##### 3.1.2.6 Guilt and perceived burden

Fourteen participants experienced feelings of guilt while receiving support from their families, perceiving themselves as a burden to their loved ones.

“Seeing my wife running around taking care of everything makes me feel indebted to her. She tells me, ‘Don’t worry about it,’ and I understand what she means, but I still can’t help feeling guilty” (P12).

“I was afraid that the illness wouldn’t be completely cured, and I worried about causing trouble for my husband and daughter, becoming a burden to them” (P2).

#### 3.1.3 Limited social roles and functional capacity

Patients with advanced lung cancer undergoing chemotherapy experience a range of psychosomatic symptoms that together with the disease itself and treatment-related side effects, contribute to reduced social participation and impaired functional capacity.

##### 3.1.3.1 Decline in self-care ability

Fourteen participants reported limitations in performing activities of daily living, leading to increased dependence on others for assistance.

“Now I pay extra attention when going to the toilet. Getting up and sitting down has become difficult for me. So, I need to use handrails or rely on family members for assistance” (P17).

“After chemotherapy, I experienced numbness in my fingers, which made it hard to get dressed. My hands wouldn’t follow my intentions, and my movements became slow. I often couldn’t fasten buttons properly and needed help from my family” (P2).

##### 3.1.3.2 Changes in family roles

Nine participants described shifts in family roles, transitioning from primary caregivers or financial providers to individuals who required care and support.

“I used to handle almost all the household chores by myself, including cooking, cleaning, and daily errands. But now I’ve become the idlest person in the family. Most of the time, I just sit or lie down and watch my family do the things I used to do” (P16).

“I always feel like I owe my family a lot. In the past, I could earn money and take care of them; they all relied on me. Now, not only can I not help them, but I also make them worry about me” (P10).

##### 3.1.3.3 Decline in work capacity or unemployment

Illness and treatment-related effects impaired participants’ ability to work, with six participants being compelled to take prolonged sick leave, change occupations, or discontinue employment altogether.

“I work as a finance officer in the company. This job requires a clear mind. Now my memory isn’t good. Sometimes I forget what I was doing and can’t continue working. I have taken two months of sick leave” (P2).

“After I got sick and started feeling unwell, I couldn’t continue working and eventually had to quit my previous job. For a while, I wasn’t sure what I could do. Now, I work as a game streamer, which I can manage from home at my own pace” (P15).

##### 3.1.3.4 Social avoidance or withdrawal

Sixteen participants deliberately reduced or avoided social interactions due to physical deterioration, changes in appearance, or persistent low mood.

“I’m not really willing to interact with others. I don’t want to go out and visit neighbors. When people ask why I’ve been absent, I don’t feel like explaining. I just want to stay at home” (P13).

“Back in the village, I used to chat with friends while basking in the sun. Now I rarely leave the house and don’t feel like talking to others” (P4).

##### 3.1.3.5 Perceived stigma

Eight participants expressed concerns that others would perceive them primarily as “ill” individuals, which led them to avoid social occasions and limit interactions with the outside world.

“Getting sick isn’t something to be proud of. There’s really not much to talk about. No matter how much you discuss it, the illness is still there, and nothing can change that” (P6).

“At work, I chat and laugh with colleagues every day. When they learned I was hospitalized, they said they’d come to visit me. I really didn’t want them to come. I don’t want them to think of me as sick and as a patient” (P16).

### 3.2 Symptom management strategies

#### 3.2.1 Positive approach to challenges

Older participants who had been diagnosed with advanced lung cancer for an extended period and were undergoing chemotherapy described gradually accepting the reality of their illness. These individuals reported a strong sense of agency in managing their symptoms, believing that moderate physical activity, proactive self-adjustment, and open communication with healthcare professionals could help alleviate discomfort.

“I find that chatting with my family is the most effective approach. Sometimes I chat with my daughter for a long time—time passes quickly, and it greatly eases my mind” (P2).

“Following the doctor’s advice, I take my medications, receive injections, and rest as instructed. When I feel short of breath, I sit down and practice deep breathing. When I feel irritable, I go downstairs for a walk or look out the window to get some sunlight” (P10).

“When my throat hurts, I drink more warm water, and the doctor advised me to avoid irritating foods. When my skin itches, I apply ointment, and it has improved a lot recently. When I feel distressed, I browse my phone, read the news, or walk to the corridor for some fresh air” (P11).

#### 3.2.2 Negative responses to symptoms

Fifteen participants undergoing chemotherapy for advanced lung cancer reported pessimistic or negative attitudes toward their symptoms, often adopting coping behaviors characterized by resignation, passive endurance, or emotional suppression.

“Sometimes when I hear that some fellow patients didn’t have any effect after treatment, I get scared. They spent so much money on treatment and then suddenly died. I thought the treatment was meaningless and that it might be better not to treat at all, just go home and die” (P13).

“I never told the doctors or nurses about my anxiety or restlessness. They’re already so busy and exhausted with their work. Treating my physical illness felt like more than enough and bringing up my emotional pain seemed inappropriate” (P7).

“What can I do? I just keep everything bottled up. I can’t take my frustration out on my wife; she’s exhausted enough already. I can only direct my anger at myself, sometimes even hitting my own leg out of frustration” (P16).

### 3.3 Symptom outcomes

#### 3.3.1 Persistent psychological burden and disease-related uncertainty

Twelve participants were unable to effectively manage their symptoms or relieve internal stress, resulting in persistent anxiety and a prolonged psychological burden.

##### 3.3.1.1 Persistent emotional distress

Six participants reported enduring psychological stress throughout the treatment process, often finding it difficult to achieve complete relief.

“(With a bitter smile) It’s only a temporary fix, like trying to patch a big hole with a small piece of cloth. It doesn’t really address the root of the problem. I know it helps a little in the moment, but deep down, I’ve always known that my illness won’t get better” (P1).

“(Eyes brimming with tears) Sigh, I’m just barely holding on. Sometimes I am able to think things through and carry on with daily activities such as eating and drinking, but at other times, when these emotions arise, I find it difficult to stop my thoughts from spiraling into pessimism” (P2).

##### 3.3.1.2 Uncertainty about disease progression and prognosis

Eleven participants reported persistent uncertainty regarding the future course of their illness and their living conditions.

“I’m afraid I won’t be cured. Sometimes I wake up in the middle of the night, and my mind starts to wander. I think about my grandson being so young and wonder what would happen if I died someday. The more I think about it, the more distressed I become” (P13).

“I don’t have a clear idea either. Has the disease been controlled? How many more years can I live? I don’t dare to ask, and I’m afraid of the answer” (P4).

#### 3.3.2 Reconstructing psychosomatic adaptation and self-management abilities

As treatment progressed, sixteen participants gradually developed adaptive strategies for managing their symptoms and enhancing their psychosomatic resilience.

##### 3.3.2.1 Acceptance and integration of illness

Fourteen participants gradually came to accept the reality of their illness and incorporated it into their life experience.

“Later, I realized that my instability only made my family more anxious. Now I strive to remain steady. I remind myself every day, living is winning” (P12).

“I gradually accepted it. Everyone has to go through this stage. Eventually, everyone will get sick. I just hope I can suffer less. Now that I’ve understood this, I’m actually calmer than before” (P9).

##### 3.3.2.2 Developing personalized management strategies

Twelve participants developed individualized routines and strategies based on their own experiences to maintain relatively stable daily rhythms.

“I maintain a normal diet every day, follow a regular schedule, engage in appropriate activities, take medication as prescribed by the doctor, undergo regular check-ups, come for chemotherapy on time when needed, and maintain a very regular daily life. This is quite good” (P10).

“I feel much better than before. At first, I didn’t understand certain things, but now I know what I can and cannot eat, and I can monitor my blood glucose myself. My mindset has improved. I can actively adjust, follow the doctor’s advice, and avoid unnecessary worry” (P9).

##### 3.3.2.3 Increasing self-efficacy and sense of control

Eight participants reported that, through accumulated experience in managing their symptoms, they had developed greater confidence in their ability to cope with illness.

“At my age, I’ve been through a lot, and my mindset has adjusted relatively quickly. Now my emotions are relatively calm. I often tell myself that anxiety and fear don’t solve practical problems. Instead, they may even make the situation worse” (P3).

“My mindset has improved as well. I can actively regulate myself, follow the doctor’s advice, and avoid random worrying. Sometimes I even encourage the patients next door, telling them not to focus constantly on their illness; the more you think about it, the worse it gets. Cherish the present” (P9).

#### 3.3.3 Seeking professional medical assistance and psychological support

When self-management proved insufficient or symptoms worsened, sixteen participants sought guidance and psychological support from healthcare professionals.

“I hope the doctor can tell me more about the changes in my condition. Nothing too technical, just enough to keep me informed. I also hope they can teach us how to relieve uncomfortable symptoms, like guidance on exercise and diet” (P1).

“When I feel upset, I just talk to the doctor or a nurse for a while. Sometimes, just a few comforting words from them can make me feel much better” (P16).

### 3.4 Influencing factors

#### 3.4.1 Individual factors

Individual differences such as age, personality traits, coping styles, and past life experiences, influence patients’ subjective symptom perception and responses.

“(Sighs) At my age, if I can endure it myself, I just do. Telling the children too much only makes them worry unnecessarily” (P4).

“I’m an independent person. If I can handle something on my own, I never bother others. That’s been my habit my whole life” (P3).

#### 3.4.2 Disease and treatment factors

The disease, treatment regimen, and chemotherapy-related side effects all exert varying degrees of influence on patients’ symptom experiences and coping strategies.

“The doctor said my condition is much better than that of other patients. Although I am also at advanced stage, so far, no metastasis has been found in any part of my body except the lungs” (P13).

“During earlier chemotherapy sessions, I needed repeated catheter insertions in my neck, which were quite painful. Now that I have a peripherally inserted central catheter (PICC), it’s much less uncomfortable, and the pain has decreased significantly” (P15).

“After chemotherapy, I felt nauseated and had no appetite. Sometimes I forced myself to eat a little, but it was extremely uncomfortable. I could only manage a few bites before I had to stop” (P8).

#### 3.4.3 Family and social environmental factors

Family and social contexts exert a substantial influence on patients’ psychosomatic symptom experiences and coping patterns. Adequate emotional, practical, and financial support from family members facilitates the adoption of positive coping strategies and enhances treatment engagement. In contrast, insufficient communication, perceived lack of understanding, caregiving strain, and economic pressure may intensify psychological distress and contribute to emotional suppression.

“(Smiling slightly) Yes, my husband is much more attentive than I am. He asks me every day how I’m feeling, and if it weren’t for him, I probably wouldn’t even want to come to the hospital for treatment” (P13).

“I try to talk to my wife, but she just sighs. I can’t talk to my son either—he works out of town, and I don’t want to worry about him. So, I mostly keep everything bottled up inside” (P11).

“Financially, there’s no real concern. My wife and I have a sufficient pension to cover my expenses, and the medications are affordable” (P12).

#### 3.4.4 Cultural factors

Cultural beliefs and values shape patients’ perceptions of illness and their approaches to symptom expression and coping. Influenced by traditional Chinese cultural norms that emphasize endurance, self-reliance, and emotional restraint, some patients perceive tolerating suffering as a personal responsibility.

“Our generation was taught by our parents from a young age to be hardworking and solve problems on our own. We were expected to take responsibility for our lives and not rely too much on others. Even now, facing the illness (lung cancer), I still feel that same sense of self-reliance, trying to manage things by myself as much as I can” (P6).

“I’ve always dealt with things by myself. Telling others would only make them worry, and they wouldn’t be able to help anyway. There’s no point in troubling others; some things just have to be endured alone” (P9).

## 4 Discussion

### 4.1 Psychosomatic symptom experiences of patients with advanced lung cancer undergoing chemotherapy

Patients with advanced lung cancer undergoing chemotherapy experience multiple symptom disturbances, consistent with previous research findings (4, 8, 9). In this study, patients undergoing chemotherapy for advanced lung cancer reported multiple physical symptom experiences throughout their disease and treatment, including respiratory symptoms, pain, fatigue, sleep disturbances, and gastrointestinal discomfort. These physical symptoms were reported to affect patients’ daily activities and quality of life. Long-term physical discomfort increases patients’ physical burden and was accompanied by heightened negative emotional experiences. Some patients reported treatment-related physical changes, such as hair loss and weight loss, which affected both their physical sensations and self-image. Furthermore, this study found that the intensity of patients’ symptom experiences appeared to vary depending on the chemotherapy cycle and individual tolerance to the drugs. Therefore, in clinical care, it is important to strengthen the dynamic assessment and monitoring of patients’ symptom changes (21), through health education (16), patients can understanding the progression of their symptoms could help alleviate related distress to some extent.

In addition to physical symptoms, patients often reported multiple psychological symptoms throughout the course of their disease and treatment, including anxiety, fear, depression, and uncertainty about the future, consistent with the findings of Cruz-Castellanos et al. (36). Healthcare providers can use music therapy (19) to help divert patients’ attention and reduce feelings of anxiety and depression. The accumulation and internalization of psychological symptoms was observed to further exacerbate the perception of physical discomfort, creating a reciprocal interaction that increases the overall symptom burden (37). In this study, some patients experienced substantial psychological burden following a diagnosis of advanced lung cancer, which is consistent with the findings of previous research (38). This may be related to the psychological impact of receiving an advanced cancer diagnosis, the complexity of the treatment process, and the uncertainty surrounding disease prognosis. In addition, chemotherapy-related adverse reactions and changes in physical appearance may increase patients’ psychological burden. Long-term psychological stress may intensify patients’ perception of physical discomfort, thereby creating an interaction between psychosomatic symptoms. Therefore, during the process of symptom management, healthcare providers should pay close attention to patients’ emotional changes and psychological needs. Through personalized psychological support and health education, patients can be helped to gradually adapt to the changes brought about by the disease (39). Furthermore, based on patients’ physical tolerance, they may also be encouraged to engage in appropriate exercises, such as Baduanjin and resistance training (20), which can help alleviate negative emotions.

This study also found that disease- and treatment-related symptoms affected patients’ social roles and daily functioning. Some patients reduced their social activities due to decreased physical strength or the increased burden of treatment, and their family and occupational roles changed accordingly. Therefore, during the symptom management process, medical staff should not only pay attention to patients’ psychosomatic symptoms but also consider the impact of the disease on their social functioning and role adaptation. Managing cancer and living meaningfully therapy (17) can be adopted to guide patients in reconstructing their self-identity and adapting to identity changes following disease acceptance. In addition, mobile healthcare platforms (22) can be utilized to provide dynamic and diverse education on symptom management and signs of disease progression, thereby improving patients’ disease awareness and reducing the subjective burden associated with their symptom experiences.

### 4.2 Symptom management strategies for psychosomatic symptoms in patients with advanced lung cancer undergoing chemotherapy

In this study, patients with advanced lung cancer undergoing chemotherapy reported both positive and negative coping strategies for managing psychosomatic symptoms, consistent with the findings of Chirico et al. (11). Some patients actively addressed symptom distress by adjusting their lifestyle, engaging in moderate physical activity, diverting their attention, and communicating with family members or medical staff. To some extent, these strategies helped alleviate physical discomfort and emotional stress. Studies have shown that patients who employ positive coping strategies are likely to alleviate discomfort by adjusting their daily routines, seeking support from family, society, or healthcare professionals, and generally maintain more stable emotional states (12). In this study, some patients with a longer time since diagnosis gradually accepted the reality of their disease, developed a stronger understanding and ability to regulate their symptoms, and were therefore more inclined to adopt active strategies and self-management strategies to cope with symptom distress. Some patients adopted negative coping strategies when facing symptom-related distress. Those who relied on such strategies were prone to behaviors including denial and self-blame, social isolation, and cognitive or behavioral avoidance, which further exacerbated their emotional distress (13). During interviews, some patients exhibited behaviors such as resistance to treatment, self-withdrawal, avoidance, and neglect of symptoms when confronted with their psychosomatic experiences. This may be related to a lack of effective coping strategies for managing symptoms like pain, coughing, and nausea, which can lead to restlessness, anxiety, and even symptom exaggeration. Patients may catastrophize their symptoms, subsequently engaging in avoidance or other negative coping behaviors. Some male patients, those with higher levels of social support, and patients with stage III disease were more likely to adopt an active coping style, which may be related to higher self-efficacy and positive disease cognition, consistent with the findings of Meneguin et al. (14). Other studies (15, 40) have shown that lower self-efficacy, heightened disease perception, and elevated psychological stress can prompt patients to adopt a passive coping style.

Some patients, although aware of the importance of symptom management, find it difficult to take active coping actions due to physical decline, ongoing psychological stress, or low self-efficacy. Therefore, when assessing patients’ symptoms, medical staff should simultaneously identify their coping styles and provide targeted support. In addition to medication guidance, interventions such as magnanimous therapy (18), nurse-led and web-based psychoeducational programs (16), and virtual reality-supported acceptance and commitment therapy (23) can help patients correct misperceptions, reconstruct cognitive frameworks, and enhance self-efficacy. Additionally, peer education and experience sharing may strengthen coping confidence, reinforce proactive responses, reduce catastrophic interpretations of symptoms, and promote the development of effective self-management strategies.

### 4.3 Outcomes of symptom management in patients with advanced lung cancer undergoing chemotherapy

This study found that patients with advanced lung cancer undergoing chemotherapy exhibited diverse symptom outcomes. Some patients experienced persistent psychological stress and uncertainty regarding disease progression throughout the treatment process. Ongoing worry and emotional burden may lead to negative outcomes, affecting patients’ quality of life, consistent with the findings of Akechi et al. (41). Although some patients were able to temporarily alleviate their emotions through short-term coping strategies, they still struggle to fully eliminate concerns about disease progression and future living conditions. When patients adopt negative coping strategies such as avoidance, repression, or catastrophizing, their symptoms and emotional stress are more likely to form a continuous cycle, thereby reducing the long-term effectiveness of symptom management. In contrast, some patients, influenced by disease acceptance, self-adjustment, and external support, gradually rebuild their symptom management skills and sense of life control, consistent with the findings of Luo et al. (6).

In this study, some patients gradually developed individualized symptom management strategies based on their understanding of the disease and integration of their personal treatment experiences. With guidance from medical staff and through personal practice, they learned to recognize changes in symptoms, adjust coping strategies accordingly, and enhance both self-efficacy and their sense of control over daily life,consistent with the findings of previous studies (16, 42). This may be because the patient adopts an active coping approach, characterized by taking initiative and seeking support. Interaction with caregivers helps them gain a deeper understanding of the patient’s ability and confidence in self-managing symptoms and assists the patient in developing personalized symptom management strategies, thereby improving symptom outcomes.

This study also found that when patients have limited self-regulation abilities or experience worsening symptoms, they tend to seek professional guidance and emotional support from healthcare providers. Zhang et al. (43) found that comprehensive symptom management effectively alleviates patients’ anxiety and depression while improving quality of life. When nursing staff continuously monitor patients’ symptom experiences and provide clear, understandable explanations and guidance, patients are more likely to gain a sense of security and support. At the same time, family members play a crucial role in daily care and emotional support, and the broader social support network further expands patients’ coping resources. In future clinical practice, medical staff should prioritize patients’ symptom experiences. Through systematic assessment, dynamic follow-up, multidisciplinary collaboration, and the application of emerging technologies and evidence-based interventions, they can help patients maintain relative stability in physical and psychological status and overall quality of life across the disease trajectory.

### 4.4 Multiple factors influencing the management of psychosomatic symptoms in patients with advanced lung cancer undergoing chemotherapy

This study found that the psychosomatic symptoms of patients with advanced lung cancer undergoing chemotherapy are influenced by multiple factors, including individual physiological characteristics, prior disease management experience, family structure and social environment, economic pressures, treatment regimen and cultural background, consistent with the findings of de Mol et al. (44). These factors may affect patients’ symptom experiences, coping strategies, and the effectiveness of symptom management. During the interviews, some older adult and widowed patients reported that when facing symptom distress, they considered factors such as not wanting to burden their children or trouble medical staff. As a result, these patients were more likely to cope with symptoms alone, and their symptom experiences were more pronounced. In this study, some patients reported a decline in physical condition and weakened immunity following the COVID-19 pandemic, consistent with the findings of Passaro et al. (45). Some patients reported that a noisy hospital environment and poor sleep negatively affected their mental well-being. In future clinical practice, healthcare professionals should aim to centralize treatment, provide comprehensive admission education and explanations to patients and their families, reduce or standardize visiting hours, and ensure patients have sufficient rest. Li et al. (46) found that a lower symptom burden in patients is associated with greater benefits for family caregivers. This highlights the importance of involving family caregivers in symptom management and implementing strategies to actively engage them, thereby reducing patients’ symptom burden and enhancing caregivers’ perceived benefits.

In the context of traditional Chinese culture, some patients view strength, endurance, and self-reliance as personal responsibilities. As a result, when facing illness and symptom-related distress, they may hide their pain or suppress emotional expression. This cultural orientation can influence patients’ subjective perception and reporting of symptoms, leading some to underestimate or delay communicating their discomfort, which may affect timely access to medical care and symptom management. These findings are consistent with Wang et al. (47). Therefore, in clinical practice, it is essential to consider the impact of cultural background on patients’ health behaviors. By using culturally sensitive communication strategies, healthcare providers can encourage patients to express physical discomfort and emotional experiences, while integrating patients’ cultural values into health education and symptom management guidance, thereby promoting more active patient participation in the symptom management process.

In addition, the assessment and formulation of individualized psychosomatic care plans should consider patients’ personal characteristics, family functioning, social support systems, and cultural values. For patients with limited family support, professional care and follow-up management should be strengthened. Middle-aged patients experiencing heavy role-related pressures should receive targeted stress relief and psychological support. Furthermore, a gentle and supportive communication approach should be adopted to encourage patients to express their needs, address multiple influencing factors, and ultimately reduce their psychosomatic symptom burden.

## 5 Limitations

Several limitations should be acknowledged in this study. First, participants were recruited from a single tertiary hospital in one province of China, so the sample may reflect the characteristics of patients receiving care within this specific institutional and regional context. Therefore, the findings should be interpreted with caution when considering their applicability to patients in other regions, healthcare settings, or cultural contexts, which may limit the broader generalizability of the results. Second, researchers’ prior knowledge and assumptions could have influenced data interpretation. To enhance the credibility and applicability of future research, multicenter studies across diverse regions are recommended to better generalize findings to all patients undergoing chemotherapy for advanced lung cancer. Additionally, longitudinal study designs could be employed to examine the temporal evolution of symptom experiences, thereby providing a more comprehensive understanding of psychosomatic symptom trajectories in this patient population.

## 6 Conclusion

This study conducted qualitative interviews with 17 patients undergoing chemotherapy for advanced lung cancer, guided by the SMT. It explored patients’ authentic experiences throughout the disease and treatment process and identified four major themes: symptom experiences, symptom management strategies, outcomes and influencing factors. It is suggested that medical staff should pay close attention to patients’ symptom experiences. By accurately identifying symptom fluctuations, providing individualized education and psychological support, and optimizing family and social support systems, these approaches can help improve patients’ psychosomatic symptom experiences, promote positive coping strategies, and strengthen symptom monitoring and guidance.

Furthermore, the findings of this study provide a reference for future quantitative research. Subsequent studies could build upon existing psychosomatic symptom assessment tools, such as validating the reliability and applicability of the psychosomatic symptom sale (PSSS) (48) among patients undergoing chemotherapy for advanced lung cancer. Based on the unique symptom experiences of this population, researchers could also optimize or develop more targeted measurement tools. Additionally, future research could integrate SMT to design comprehensive psychosomatic symptom management interventions. Longitudinal studies could then explore the trajectory of psychosomatic symptom changes in patients undergoing chemotherapy for advanced lung cancer. Such efforts may help reduce patients’ psychological and physical distress, improve quality of life, and ultimately contribute to better long-term outcomes.

## Data Availability

The original contributions presented in the study are included in the article/Supplementary material, further inquiries can be directed to the corresponding author.
