# Supplementary material for: Experiences of psychosomatic symptoms and self-management strategies among patients with advanced lung cancer undergoing chemotherapy: a qualitative study based on symptom management theory
Source: Front Public Health. 2026 Mar 27;14:1807299. doi: 10.3389/fpubh.2026.1807299 (PMC13066165; doi:10.3389/fpubh.2026.1807299)
Supplement: Supplementary file 1 [file Table_1.docx]

Supplementary Material

1. **Consolidated criteria for reporting qualitative studies (COREQ): 32-item checklist**

Developed from:

Tong, A., Sainsbury, P., & Craig, J. (2007). Consolidated criteria for reporting qualitative research (COREQ): A 32-item checklist for interviews and focus groups. *International Journal for Quality in Health Care, 19*(6), 349–357. https://doi.org/10.1093/intqhc/mzm042

| **No. Item** | **Guide questions/description** | **Answer** | **Reported on Page #** |
| --- | --- | --- | --- |
| Domain 1: Research team and reﬂexivity |  |  |  |
| Personal Characteristics |  |  |  |
| 1.Interviewer*/*facilitator | Which author*/*s conducted the interview or focus group? | BX | Page 4  2.5 Data analysis |
| 2. Credentials | What were the researcher’s credentials? E.g. PhD, MD | Three postgraduate students in nursing, and one PhD | Page 4  2.5 Data analysis |
| 3. Occupation | What was their occupation at the time of the study? | Three postgraduate students in nursing, and one is a professor in nursing school | Page 4  2.5 Data analysis |
| 4. Gender | Was the researcher male or female? | Female | Page 4  2.5 Data analysis |
| 5.Experiencing and training | What experience or training did the researcher have? | All researchers have received training in qualitative research methods | Page 4  2.5 Data analysis |
| Relationship with participants |  |  |  |
| 6. Relationship established | Was a relationship established prior to study commencement? | No | Page 4  2.7 Rigor and reflexivity |
| 7. Participant knowledge of the interviewer | What did the participants know about the researcher? e.g. personal goals, reasons for doing the research | The research aims, the reasons for conducting the study, and what we hope to improve for this population. | Page 3  2.4 Data collection |
| 8.Interviewer characteristics | What characteristics were reported about the interviewer/facilitator? e.g. Bias, assumptions, reasons and interests in the research topic | We avoided making assumptions before collecting and analyzing the interview data. | Page 4  2.7 Rigor and reflexivity |
| Doman 2: study design |  |  |  |
| Theoretical framework |  |  |  |
| 9.Methodological orientation and Theory | What methodological orientation was stated to underpin the study? e.g. grounded theory, discourse analysis, ethnography, phenomenology, content analysis | Content analysis | Page 4  2.5 Data analysis |
| Participant selection |  |  |  |
| 10.Sampling | How were participants selected? e.g. purposive, convenience, consecutive, snowball | Purposive | Page 3  2.2 Study setting and recruitment |
| 11.Method of approach | How were participants approached? e.g. face-to-face, telephone, mail, email | Face-to-face | Page 3  2.4 Data collection |
| 12.Sample size | How many participants were in the study? | 17 participants | Page 4  3 Results |
| 13. Non-participation | How many people refused to participate or dropped out? Reasons? | No | Page 3  2.4 Data collection |
| Setting |  |  |  |
| 14.Setting of data collection | Where was the data collected? e.g. home, clinic, workplace | The data collected in a quiet and comfortable demonstration classroom within the oncology department | Page 3  2.4 Data collection |
| 15. Presence of non-participants | Was anyone else present besides the participants and researchers? | No | Page 3  2.4 Data collection |
| 16.Description of sample | What are the important characteristics of the sample? *e.g. demographic data, date* | Please see Table 1 | Table 1 |
| Data collection |  |  |  |
| 17.Interview guide | Were questions, prompts, guides provided by the authors? Was it pilot tested? | Yes. Yes | Page 3  2.3 Interview outline |
| 18. Repeat interviews | Were repeat interviews carried out? If yes, how many? | No | Page 3  2.4 Data collection |
| 19.Audio/visual recording | Did the research use audio or visual recording to collect the data? | Yes. Audio recording | Page 3  2.4 Data collection |
| 20.Field notes | Were ﬁeld notes made during and/or after the interview or focus group? | Yes | Page 3  2.4 Data collection |
| 21. Duration | What was the duration of the interviews or focus group? | About 30-45 mins each | Page 3  2.4 Data collection |
| 22. Data saturation | Was data saturation discussed? | Yes | Page 3  2.2 Study setting and recruitment |
| 23. Transcripts returned | Were transcripts returned to participants for comment and/or correction? | Yes | Page 4  2.5 Data analysis |
| Domain 3: analysis and fingings |  |  |  |
| Data analysis |  |  |  |
| 24. Number of data coders | How many data coders coded the data? | 2 coders | Page 4  2.5 Data analysis |
| 25.Description of the coding tree | Did authors provide a description of the coding tree? | No | N/A |
| 26. Derivation of themes | Were themes identiﬁed in advance or derived from the data? | Advance | Page 4  2.5 Data analysis |
| 27. Software | What software, if applicable, was used to manage the data? | NVivo 15.0 software | Page 4  2.5 Data analysis |
| 28.Participant checking | Did participants provide feedback on the ﬁndings? | No | N/A |
| Reporting |  |  |  |
| 29. Quotations presented | Were participant quotations presented to illustrate the themes */* ﬁndings? Was each quotation identidied? E.g. participant number | Yes. Yes | Page 4-10  3 Results |
| 30. Data and findings consistent | Was there consistency between the data presented and the ﬁndings? | Yes | Page 4-10  3 Results |
| 31.Clarity of major themes | Were major themes clearly presented in the ﬁndings? | Yes | Page 4-10  3 Results |
| 32.Clarity of minor themes | Is there a description of diverse cases or discussion of minor themes? | Yes | Page 10-12  4 Discussion |

N/A: not applicable.
